# Biopsy-Confirmed Acute Interstitial Nephritis in Patients Treated with Immune Checkpoint Inhibitors: A Single-Center Retrospective Case Series

**DOI:** 10.3390/antib15040065

**Published:** 2026-07-27

**Authors:** Ioannis Ogrotis, Konstantinos Drouzas, Evangelia Pantzopoulou, Petros Nikolopoulos, Ioannis Kotsantis, Amanda Psyrri, George Liapis, Sophia Lionaki

**Affiliations:** 1 Department of Clinical & Interventional Nephrology, 2nd Propaedeutic Clinic of Internal Medicine, Attikon University Hospital, School of Medicine, National and Kapodistrian University of Athens, Rimini 1, Chaidari, 12461 Athens, Greece; iogrotis@auth.com (I.O.); drouziboy@hotmail.com (K.D.); evapantz@hotmail.com (E.P.); nikolopoulospetros@gmail.com (P.N.); 2 Oncology Department, 2nd Propaedeutic Clinic of Internal Medicine, Attikon University Hospital, National and Kapodistrian University of Athens, Rimini 1, Chaidari, 12461 Athens, Greece; ikotsantis@med.uoa.gr (I.K.); psyrrioncology@gmail.com (A.P.); 3 1st Department of Pathology, Medical School, National and Kapodistrian University of Athens and Laikon Hospital, 11527 Athens, Greece; gliapis@gmail.com

**Keywords:** immune checkpoint inhibitors, acute interstitial nephritis, acute kidney injury, renal biopsy, immune-related adverse events, ICI-associated nephrotoxicity, onconephrology, renal recovery, corticosteroids

## Abstract

**Background/Objectives**: Acute interstitial nephritis (AIN) is a cause of acute kidney injury (AKI) during immune checkpoint inhibitor (ICI) therapy, but biopsy-confirmed data are limited. We estimated institutional proportions of biopsy-confirmed AIN clinically attributed to ICI exposure (ICI-AIN) and characterized its presentation, pathology, treatment, and renal outcomes. **Methods**: We retrospectively reviewed native renal biopsies performed at Attikon University Hospital from February 2021 through December 2025. Cases were included when ICI exposure preceded AKI and the treating nephrology team documented clinicopathologic attribution to ICI exposure. **Results**: AIN was identified in 15 of 339 native renal biopsies; 12 cases were attributed to ICI exposure, representing 3.5% of native renal biopsies and 0.8% of 1472 unique ICI-treated patients. Median serum creatinine increased from 1.05 mg/dL at baseline to 3.50 mg/dL at biopsy assessment. During the AKI episode, 10 patients (83.3%) met Kidney Disease: Improving Global Outcomes (KDIGO) criteria for stage 3 AKI. All patients had pyuria, negative urine cultures, and subnephrotic proteinuria. Hematuria and peripheral eosinophilia occurred in four and two patients, respectively. All patients received corticosteroids, and none required kidney replacement therapy. Complete, partial, and absent recovery occurred in eight, two, and two patients, respectively; under the stricter baseline-relative definition, the corresponding numbers were five, five, and two. **Conclusions**: Biopsy-confirmed ICI-AIN was infrequently detected. Severe AKI was common, urinary findings were nonspecific, and residual renal dysfunction frequently persisted after corticosteroid treatment.

## 1. Introduction

Immune checkpoint inhibitors (ICIs) targeting cytotoxic T-lymphocyte-associated antigen 4 (CTLA-4), programmed cell death protein 1 (PD-1), and programmed death-ligand 1 (PD-L1) have improved outcomes across multiple malignancies by enhancing antitumor T-cell activity and producing durable clinical responses [1]. However, this immune activation can extend beyond the tumor microenvironment and cause immune-related adverse events that may affect virtually any organ system [2].

Acute kidney injury (AKI) is increasingly recognized in patients receiving ICIs [3]. Importantly, AKI that develops during ICI therapy should not automatically be attributed to immune-mediated ICI nephrotoxicity or classified as immune checkpoint inhibitor-associated AKI (ICI-AKI). In observational cohorts, creatinine-defined AKI occurs in approximately 15–20% of ICI-treated patients, whereas AKI adjudicated as likely ICI-associated occurs in approximately 3–5% [3,4]. A recent meta-analysis reported a pooled all-cause AKI rate of 7.4% across all included studies, 14.6% in retrospective studies, and 3.2% for ICI-related AKI [5]. This distinction is clinically important because patients with cancer frequently have alternative or concurrent causes of renal injury, including hemodynamic disturbances, infection, urinary obstruction, iodinated contrast exposure, chemotherapy-related nephrotoxicity, and concomitant use of medications such as proton pump inhibitors, nonsteroidal anti-inflammatory drugs, and selected antibiotics [3,5,6].

Among biopsy-confirmed renal lesions attributed to ICI therapy, acute tubulointerstitial nephritis—commonly termed acute interstitial nephritis (AIN) in clinical practice—is the predominant histopathologic pattern [7,8]. In the original clinicopathologic series by Cortazar et al. [7], acute tubulointerstitial nephritis was present in 12 of 13 biopsied patients with checkpoint inhibitor-associated AKI. AIN was also the dominant lesion among biopsied patients in a larger international cohort of ICI-AKI [9]. However, acute tubular injury and a range of glomerular diseases have also been reported [10,11]. Thus, although AIN is the most characteristic biopsy-confirmed renal lesion associated with checkpoint blockade, suspected ICI-AKI should not be presumed to represent AIN without consideration of alternative or concurrent lesions.

Establishing both the diagnosis of AIN and its attribution to ICI therapy remains challenging in clinical practice [6,12]. Classic hypersensitivity manifestations are usually absent, and noninvasive findings such as sterile pyuria, low-grade proteinuria, microscopic hematuria, and peripheral eosinophilia are insufficiently specific to establish the diagnosis [8,12]. The timing of AKI relative to ICI initiation, the number of treatment cycles, and the interval since the last ICI dose may inform attribution but are not diagnostic, because ICI-related AKI can occur shortly after treatment initiation, after prolonged exposure, or even after treatment discontinuation [8,9]. Concomitant use of medications classically associated with AIN—particularly proton pump inhibitors, nonsteroidal anti-inflammatory drugs, and selected antibiotics—further complicates causal attribution [5,8]. One proposed mechanism is that checkpoint blockade unmasks or amplifies drug-specific or self-reactive T-cell responses in susceptible patients [6]. Accordingly, careful medication review, systematic assessment of competing AKI etiologies, and detailed reconstruction of ICI exposure chronology are central to the diagnostic evaluation.

These diagnostic uncertainties have direct management implications. Temporary interruption or permanent discontinuation of immunotherapy may compromise oncologic disease control, whereas empiric high-dose corticosteroid treatment may expose patients with non-immune-mediated renal lesions to avoidable toxicity. Renal biopsy is not required in every suspected case, and oncology- and nephrology-oriented approaches differ in the emphasis they place on histopathologic confirmation. Nevertheless, biopsy provides the most definitive diagnostic information when alternative etiologies are plausible, urinary findings are atypical, AKI is severe or persistent, or renal and oncologic management decisions carry substantial consequences [12,13,14]. Histopathologic confirmation can help avoid unnecessary ICI discontinuation or corticosteroid treatment when AIN is absent and can support timely immunosuppression when AIN is confirmed [12,14].

Despite growing recognition of ICI-associated renal injury, biopsy-confirmed institutional data remain scarce, and the frequency of biopsy-confirmed AIN clinically attributed to ICI exposure in routine practice remains poorly defined. We therefore aimed to characterize the demographic and oncologic features, clinical presentation, laboratory and histopathologic findings, treatment, and renal outcomes of patients with biopsy-confirmed AIN clinically attributed to ICI exposure (hereafter termed ICI-AIN), and to estimate the institutional proportions of biopsy-confirmed ICI-AIN among native renal biopsies and unique ICI-treated patients during the study period.

## 2. Materials and Methods

### 2.1. Study Design

We conducted a single-center, retrospective observational case series of patients with biopsy-confirmed AIN clinically attributed to ICI exposure (hereafter termed ICI-AIN). The study included cases diagnosed at Attikon University Hospital, a tertiary academic center in Athens, Greece, from February 2021 through December 2025. The study was conducted in accordance with the Declaration of Helsinki. The Scientific Council of the University General Hospital “Attikon” issued an exemption letter stating that approval by the Bioethics Committee of the University General Hospital “Attikon”–General Hospital of Western Attica “Agia Varvara” (Headquarters Unit, University General Hospital “Attikon”) was not required for this retrospective study. The letter further stated that written informed consent was documented for all included patients.

### 2.2. Case Ascertainment and Cohort Eligibility

Eligible cases were identified through the institutional renal pathology archive. All native renal biopsies performed during the study period were screened for histopathologic findings consistent with acute tubulointerstitial nephritis, clinically referred to as AIN. Cases of biopsy-confirmed AIN were then cross-referenced with the institutional oncology registry and medical records to identify patients with documented exposure to ICIs before the onset of AKI and a documented clinicopathologic attribution to ICI exposure by the treating nephrology team.

Adult patients were eligible for inclusion if they had undergone a native renal biopsy during the study period, had histopathologic findings consistent with AIN, had documented ICI exposure before AKI onset, and had a treating-team clinicopathologic assessment attributing AIN to ICI exposure after consideration of competing causes. Renal transplant recipients and patients receiving maintenance kidney replacement therapy before ICI initiation were excluded.

We documented the case-ascertainment process, including the number of native renal biopsies screened, the number of biopsy-confirmed AIN cases identified, the number of these cases with documented ICI exposure before AKI onset and a clinical attribution to ICI exposure, and the final number of patients included in the ICI-AIN cohort.

### 2.3. Clinical Attribution to ICI Exposure and Clinical Data Collection

The histopathologic diagnosis of AIN and its clinical attribution to ICI exposure were considered separate components of case classification. The diagnosis of AIN was based on the final renal pathology report. Inclusion in the ICI-AIN cohort required documented ICI treatment before AKI onset and a clinicopathologic attribution to ICI exposure recorded by the treating nephrology team after a review of the temporal context, concomitant medications, and competing causes of AKI [12,14]. Competing etiologies considered included volume depletion or hemodynamic injury, infection, urinary obstruction, recent iodinated contrast exposure, concomitant nephrotoxic chemotherapy, antibiotics, nonsteroidal anti-inflammatory drugs, proton pump inhibitors, and other medications associated with drug-induced AIN, together with urinalysis, urine-culture results, renal imaging when available, and the overall clinical course. Thus, biopsy established the diagnosis of AIN, whereas attribution to ICI exposure remained a clinical judgment rather than a histology-specific determination. The study did not perform an independent, standardized, or blinded causality adjudication. Qualitative temporal information available in the treating clinicians’ notes informed routine clinical attribution, but the dates and intervals were not sufficiently complete or standardized for patient-level or cohort-level reporting. Renal biopsy decisions were made during routine clinical care rather than according to a prespecified study protocol.

The indication for renal biopsy was not captured through a standardized field in the medical record. Because the contemporaneous rationale could not be assigned reliably in every patient, formal patient-level biopsy-indication categories were not retrospectively constructed. Instead, the objective clinical context surrounding biopsy was summarized using the presence of ongoing renal dysfunction at biopsy, the highest KDIGO AKI stage eventually reached during the AKI episode, urinary findings, clinical presentation, and the interval from AKI onset to biopsy.

Medical records were reviewed using a structured patient-level dataset to extract demographic, oncologic, clinical, laboratory, treatment, pathology, and follow-up data. Collected baseline variables included age, sex, baseline renal function, documented chronic kidney disease when available, malignancy type, cancer histology, ICI regimen, and concomitant medications documented as active at the time of AKI evaluation. Aggregate tables were derived from the patient-level dataset. Values that could not be confirmed in the available source record were coded as missing rather than inferred.

ICI exposure data were limited to the ICI agent or combination regimen. Detailed treatment chronology—including treatment timing and cycle count, discontinuation or rechallenge, and concurrent or recent chemotherapy—was not consistently available and was therefore not analyzed.

Medication variables included proton pump inhibitor use, H_2_-receptor antagonist use, nonsteroidal anti-inflammatory drug exposure, antibiotic exposure, and other medications classically associated with drug-induced AIN when documented as active at the time of AKI evaluation. Exposure was classified as absent only when non-use was explicitly documented; otherwise, lack of documentation was treated as unknown.

Treatment variables included corticosteroid therapy, use of intravenous methylprednisolone pulses, documented pulse dose and duration when available, oral corticosteroid therapy, additional immunosuppression, mycophenolate mofetil use, and requirement for kidney replacement therapy. Detailed patient-level data on corticosteroid initiation relative to AKI onset or renal biopsy, initial oral corticosteroid dose, taper duration, cumulative corticosteroid exposure, withdrawal of suspected AIN-associated medications, and steroid-related adverse effects were inconsistently documented and were therefore not analyzed.

Follow-up variables included serum creatinine (SCr) and estimated glomerular filtration rate (eGFR) at the last available assessment, renal recovery status under the primary and sensitivity definitions, time to the assigned primary recovery endpoint, vital status, and cause of death when available. Time to the assigned recovery endpoint was calculated from AKI onset to the first dated SCr measurement meeting the primary complete- or partial-recovery criterion. The last available assessment was not a uniform follow-up time point, and total follow-up duration from AKI onset or renal biopsy could not be consistently reconstructed; therefore, it was not summarized.

### 2.4. Definitions

Baseline SCr was defined as the most recent stable pre-AKI SCr value available in the medical record, preferably from an outpatient measurement within 3 months before AKI onset and before the diagnostic creatinine rise. When a stable outpatient value within this window was unavailable, the closest documented pre-AKI SCr value was used. Baseline eGFR < 60 mL/min/1.73 m^2^ was reported descriptively and was not treated as equivalent to a confirmed diagnosis of chronic kidney disease without evidence of chronicity.

SCr at biopsy assessment was defined as the SCr measurement obtained on the biopsy date or, when a same-day value was unavailable, the closest preceding measurement during the same AKI episode. Urinalysis, urine microscopy, urine culture, and 24-h urinary protein excretion were abstracted from measurements closest to biopsy during the active AKI episode. Values first obtained during later follow-up were not back-assigned to the diagnostic presentation.

Baseline eGFR and eGFR at the last available assessment were calculated using the CKD-EPI 2021 creatinine equation and expressed as mL/min/1.73 m^2^ [15]. These values were reported descriptively. Creatinine-based eGFR values during active AKI were not used to quantify AKI severity because eGFR equations are not validated as precise filtration estimates under non-steady-state conditions. AKI was defined according to KDIGO serum creatinine criteria. AKI stage was assigned from the highest serum creatinine value recorded during the AKI episode (peak serum creatinine), using the individual baseline serum creatinine and the applicable KDIGO serum creatinine thresholds; urine output criteria were not used because reliable hourly urine output data were not consistently available [16]. AKI onset was defined as the first date on which KDIGO serum creatinine criteria were met. Time from AKI onset to renal biopsy was calculated in days.

Peak SCr was the highest value recorded from AKI onset through recovery or, if recovery was not achieved, through the last available assessment.

Pyuria was defined as at least 5 white blood cells per high-power field. Hematuria was defined as more than 2 red blood cells per high-power field. Peripheral eosinophilia was defined as an absolute eosinophil count greater than 500 cells/µL.

Renal recovery was categorized using thresholds previously applied at 6 months after biopsy in a study of biopsy-confirmed acute interstitial nephritis [17]. In the present study, these thresholds were applied at each patient’s last available assessment rather than at a fixed time point. Complete renal recovery was defined as improvement in SCr to within 25% of baseline or to <1.4 mg/dL. Partial renal recovery was defined as a ≥50% decrease from peak SCr without meeting complete-recovery criteria; the percentage reduction was calculated as [(peak SCr − assessment SCr)/peak SCr] × 100. Absent renal recovery was defined as failure to meet complete- or partial-recovery criteria or persistent dependence on kidney replacement therapy.

For patients who achieved complete recovery, time to complete recovery was calculated from AKI onset to the first dated serum creatinine measurement meeting complete-recovery criteria. For patients who did not achieve complete recovery but met partial-recovery criteria, time to partial recovery was calculated from AKI onset to the first dated measurement meeting partial-recovery criteria. These intervals represent first documented threshold attainment rather than the exact biological time of renal recovery and may be influenced by the timing and frequency of laboratory testing. Because the absolute serum creatinine threshold may classify some patients as completely recovered despite not returning to their individual baseline renal function, a sensitivity assessment also applied the stricter criterion of return of serum creatinine to within 25% of baseline. For exploratory pathology–outcome assessment, incomplete recovery was defined as partial or absent renal recovery according to the predefined primary recovery definition.

Renal outcomes were classified at the last available assessment under the primary and sensitivity definitions described above.

### 2.5. Renal Biopsy Evaluation

Renal biopsies were performed percutaneously under ultrasound guidance. Specimens were processed for light microscopy and immunofluorescence. Electron microscopy was performed only when sufficient tissue and technical availability permitted and when clinically indicated; it was not uniformly available across the cohort. Light-microscopy evaluation included hematoxylin and eosin, periodic acid–Schiff, Jones methenamine silver, and Masson trichrome staining, as applicable. Immunofluorescence evaluation included staining for immunoglobulins, including IgG, IgA, and IgM; complement components, including C3 and C1q; κ and λ light chains; and fibrinogen, as applicable.

The histopathologic diagnosis was based on the final renal pathology report. When documented, the following descriptive variables were abstracted: the number of glomeruli sampled for light microscopy and immunofluorescence separately; the number and percentage of globally sclerosed glomeruli; the extent and severity of interstitial inflammation, tubulitis, and interstitial edema; eosinophil-rich infiltrates; granulomas; acute tubular injury; interstitial fibrosis and tubular atrophy (IFTA); arteriosclerosis or arteriolosclerosis; red and white blood cell casts; active glomerular lesions, including mesangial or endocapillary hypercellularity, segmental sclerosis, necrosis, and crescents; and the pattern, intensity, and location of immunofluorescence staining. Electron-microscopy status was classified as performed, not performed, or not reported; unavailable or unreported electron microscopy was never interpreted as a negative examination.

The term acute tubular injury was used to describe tubular epithelial damage characterized by tubular dilatation, epithelial flattening, epithelial sloughing, and regenerative epithelial changes. The term acute tubular necrosis was reserved for cases in which necrosis was explicitly reported.

For the two patients with marked microscopic hematuria (20–25 and 100 red blood cells per high-power field, respectively) and for any patient with IgA/C3 staining on immunofluorescence, the available pathology reports were specifically reviewed for evidence of active glomerular disease, including mesangial or endocapillary hypercellularity, necrosis, crescents, red blood cell casts, segmental sclerosis, and electron-dense deposits when electron microscopy had been performed.

Because this was a retrospective study, pathology variables were abstracted from routine clinical pathology reports rather than obtained through a prespecified, blinded histopathologic re-review. Standardized grading was therefore applied only when the relevant characteristics were explicitly documented. Features that were not described in a pathology report were recorded as unavailable rather than assumed to be absent, unless their absence was explicitly stated. The same rule was applied to electron microscopy: absence of a report or lack of tissue was recorded as unavailable, not as absence of ultrastructural abnormalities.

For the exploratory descriptive assessment of histopathologic chronicity and renal recovery, global glomerulosclerosis was categorized as <20% or ≥20% of sampled glomeruli, and IFTA was categorized as mild or moderate/severe. 

### 2.6. Institutional Denominators and Biopsy-Confirmed Proportion Analysis

To estimate the institutional proportions of biopsy-confirmed ICI-AIN, we calculated its frequency among all native renal biopsies performed during the study period and among all patients treated with ICIs at Attikon University Hospital during the same period.

The ICI-treated denominator was defined as the number of unique patients recorded in the institutional oncology registry who had received at least one dose of any ICI during the study period, regardless of cancer type, ICI regimen, treatment duration, or number of doses. All 12 patients with biopsy-confirmed AIN clinically attributed to ICI exposure had received at least one ICI dose at Attikon University Hospital during the study period and were included in this institutional denominator. Exact binomial 95% confidence intervals for both proportions were calculated using the Clopper–Pearson method.

These estimates were interpreted as institutional proportions of biopsy-confirmed ICI-AIN rather than incidence estimates. The denominator did not incorporate person-time, number of ICI cycles, cumulative ICI exposure, treatment duration, cancer-specific exposure, or regimen-specific exposure. Furthermore, cases that were not clinically recognized, documented, and subjected to renal biopsy—including potentially mild or transient cases—would not have been captured.

### 2.7. Statistical Analysis

Continuous variables were summarized as medians with interquartile ranges or means with standard deviations, according to their distributions. Categorical variables were summarized as counts and percentages. Given the small sample size, the analyses were primarily descriptive.

Relationships between histopathologic findings and renal outcomes were assessed descriptively and were considered hypothesis-generating. Because of the limited overall sample size and the very small subgroups, no formal inferential conclusions were drawn from these comparisons.

Missing data were not imputed. Variable-specific denominators were reported when data availability was incomplete, and variables that could not be reliably extracted were excluded from cohort-level analyses. Statistical analyses were performed using IBM SPSS Statistics for Windows, version 31.0.2.0 (IBM Corp., Armonk, NY, USA).

## 3. Results

### 3.1. Case Ascertainment and Institutional Proportions of Biopsy-Confirmed ICI-AIN

During the study period, 339 native renal biopsies were performed at Attikon University Hospital. Screening of the renal pathology archive identified biopsy-confirmed AIN in 15 patients, representing 4.4% of all native renal biopsies. Of these patients, 12 had documented ICI exposure before AKI onset and a treating-team clinicopathologic attribution to ICI exposure and constituted the final ICI-AIN cohort. Accordingly, ICI-AIN accounted for 80.0% of all biopsy-confirmed AIN cases and 3.5% of all native renal biopsies performed during the study period, with an exact 95% confidence interval of 1.8–6.1%.

During the same period, the institutional oncology registry identified 1472 unique patients who had received at least one dose of an ICI, irrespective of cancer type. All 12 patients with biopsy-confirmed AIN clinically attributed to ICI exposure had received at least one ICI dose at Attikon University Hospital during the study period and were included among these 1472 unique patients. ICI-AIN was therefore detected in 0.8% of all unique ICI-treated patients, with an exact 95% confidence interval of 0.4–1.4%.

The remaining three biopsy-confirmed AIN cases had no documented prior ICI exposure and were excluded from the ICI-AIN cohort; their etiologic attribution was outside the scope of the present analysis.

### 3.2. Baseline Demographic, Oncologic, ICI Treatment, Concomitant Medications, and Biopsy-Timing Characteristics

The ICI-AIN cohort comprised 12 adults, of whom 10 (83.3%) were men. The median age at biopsy was 70 years (IQR, 63–73; range, 51–86). Median baseline serum creatinine was 1.05 mg/dL (IQR, 0.88–1.13), and median baseline eGFR was 74 mL/min/1.73 m^2^ (IQR, 62–96). Three patients had a baseline eGFR < 60 mL/min/1.73 m^2^.

The underlying malignancy was lung cancer in 10 patients, malignant pleural mesothelioma in one patient, and esophageal adenocarcinoma in one patient. Among the 10 patients with lung cancer, adenocarcinoma was the most common histologic subtype. One patient had small-cell lung cancer, and two had non-small cell lung cancer not otherwise specified.

ICI regimens consisted of pembrolizumab in five patients, nivolumab monotherapy in one patient, nivolumab plus ipilimumab in three patients, atezolizumab in two patients, and durvalumab in one patient. Proton pump inhibitor use was documented in three patients, and four additional patients were receiving H_2_-receptor antagonists. No active nonsteroidal anti-inflammatory drug or antibiotic exposure was recorded in the available notes; however, because non-use was not systematically documented, undocumented exposure could not be excluded.

AKI was detected during routine laboratory monitoring in all 12 patients. Three patients had additional clinical manifestations at presentation: lower-extremity edema, fatigue or malaise, and epistaxis in one patient each.

Although formal biopsy indications were not documented using standardized categories, the objective clinical context surrounding biopsy was characterized. All 12 patients had ongoing renal dysfunction at the time of biopsy. During the AKI episode, 10 patients (83.3%) eventually reached KDIGO stage 3 based on peak serum creatinine, one patient (8.3%) reached stage 2, and one patient (8.3%) reached stage 1; these peak stages should not be interpreted as the AKI stage at the time of biopsy. Two patients (16.7%) had marked microscopic hematuria, with 20–25 and 100 red blood cells per high-power field, respectively. The median interval from AKI onset to biopsy was 27.5 days (IQR, 16–30; range, 5–152). One patient underwent renal biopsy 152 days after AKI onset. Biopsy was initially deferred because the patient declined the procedure at that time. During this interval, renal function remained impaired despite conservative management and evaluation of reversible and competing causes of AKI. The patient subsequently agreed to renal biopsy because renal function failed to return to baseline and diagnostic uncertainty persisted. These findings indicate that renal dysfunction was ongoing at biopsy in all patients and that most reached KDIGO stage 3 during the AKI episode, whereas marked microscopic hematuria and a prolonged diagnostic course occurred in a minority.

Baseline demographic and oncologic characteristics, ICI treatment, clinical presentation, and biopsy timing are summarized in Table 1. Individual patient-level demographic, renal, oncologic, clinical, and biopsy-timing data are provided in Table S1.

### 3.3. Renal Function, AKI Severity, and Urinary Findings at Biopsy Assessment

At biopsy assessment, median SCr was 3.50 mg/dL (IQR, 2.42–3.88). Median peak SCr was 3.95 mg/dL (IQR, 2.82–4.65). SCr, rather than creatinine-based eGFR during active AKI, was used as the principal measure of AKI severity.

KDIGO stage was assigned using the highest SCr value recorded during the AKI episode rather than the SCr at biopsy assessment. On this basis, one patient (8.3%) reached stage 1 AKI, one patient (8.3%) reached stage 2 AKI, and 10 patients (83.3%) eventually reached stage 3 AKI during the episode.

Pyuria was documented in all 12 patients, and urine cultures were negative in all 12 patients. Four patients (33.3%) had hematuria exceeding 2 red blood cells per high-power field, including two patients with marked microscopic hematuria of 20–25 and 100 red blood cells per high-power field. Urinary casts were not documented in the available records. A 24-h urinary protein measurement was available in all 12 patients; all values were subnephrotic, with a median of 210 mg/24 h (IQR, 158–309; range, 47–670). Peripheral eosinophilia was present in two patients (16.7%). Baseline renal function and diagnostic laboratory and urinary findings are summarized in Table 2.

### 3.4. Treatment and Renal Outcomes

All 12 patients received corticosteroid therapy. Ten patients received intravenous methylprednisolone pulse therapy followed by an oral methylprednisolone taper. Pulse regimens varied from 500 mg to 1 g of methylprednisolone daily for 1–3 days.

The two patients without documented pulse therapy received oral corticosteroids. One of these patients was already receiving oral corticosteroids for atezolizumab-associated pancreatitis when AKI was diagnosed. One patient also received adjunctive mycophenolate mofetil. No patient required kidney replacement therapy.

Detailed patient-level information on the timing of corticosteroid initiation relative to AKI onset or renal biopsy, the initial oral corticosteroid dose, taper duration, cumulative corticosteroid exposure, withdrawal of suspected AIN-associated medications, and corticosteroid-related adverse effects was inconsistently documented and was therefore not analyzed at the cohort level.

At the last available assessment, eight patients (66.7%) met the predefined criteria for complete renal recovery, two patients (16.7%) met the criteria for partial renal recovery, and two patients (16.7%) had absent renal recovery. The two patients with absent recovery had reductions of 42.5% and 38.8% from peak SCr, respectively, and therefore did not meet the prespecified 50% partial-recovery threshold. No patient required kidney replacement therapy; eGFR at the last available assessment ranged from 30 to 79 mL/min/1.73 m^2^.

In the sensitivity assessment in which complete recovery required serum creatinine to return to within 25% of the individual baseline value, five patients (41.7%) achieved complete recovery, five patients (41.7%) had partial recovery, and two patients (16.7%) had absent recovery. Three patients were reclassified from complete to partial recovery. Their baseline serum creatinine values ranged from 0.74 to 0.90 mg/dL, and although their final values were <1.4 mg/dL, they remained approximately 35–50% above baseline. Patients 11 and 12 remained classified as absent recovery under the sensitivity definition.

Among the eight patients who achieved complete recovery under the primary definition, the median time to complete recovery was 89.5 days (range, 31–358). Among the two patients with partial recovery, the median time to partial recovery was 60.5 days (range, 58–63). No recovery endpoint was assigned to the two patients with absent recovery because neither the complete- nor partial-recovery threshold was reached during available follow-up. These intervals represent first documented threshold attainment. Four patients died during follow-up, all from the underlying malignancy. Recovery proportions reflect status at each patient’s last available assessment rather than fixed-time recovery probabilities. Individual treatment, renal outcomes, and survival data are summarized in Table 3.

### 3.5. Renal Pathology Findings

Based on the final pathology reports, all 12 renal biopsies demonstrated findings consistent with acute interstitial nephritis. The interstitial inflammatory infiltrates were diffuse or patchy and consisted predominantly of lymphocytes, histiocytes, and plasma cells. Interstitial edema and tubulitis were described as components of the AIN pattern, but these features were not documented with complete case-level denominators, and tubulitis severity was not uniformly graded. Consistent with the prespecified abstraction approach, pathologic features not mentioned in routine reports were treated as unavailable rather than absent.

Acute tubular injury was present in 7 of 12 patients (58.3%) and was characterized by tubular dilatation, epithelial flattening, and sloughing of epithelial cells into the tubular lumens. The severity of these changes ranged from mild to marked. Regenerative epithelial changes were among the reported features but were not uniformly documented as a separate variable.

**Figure 1:**
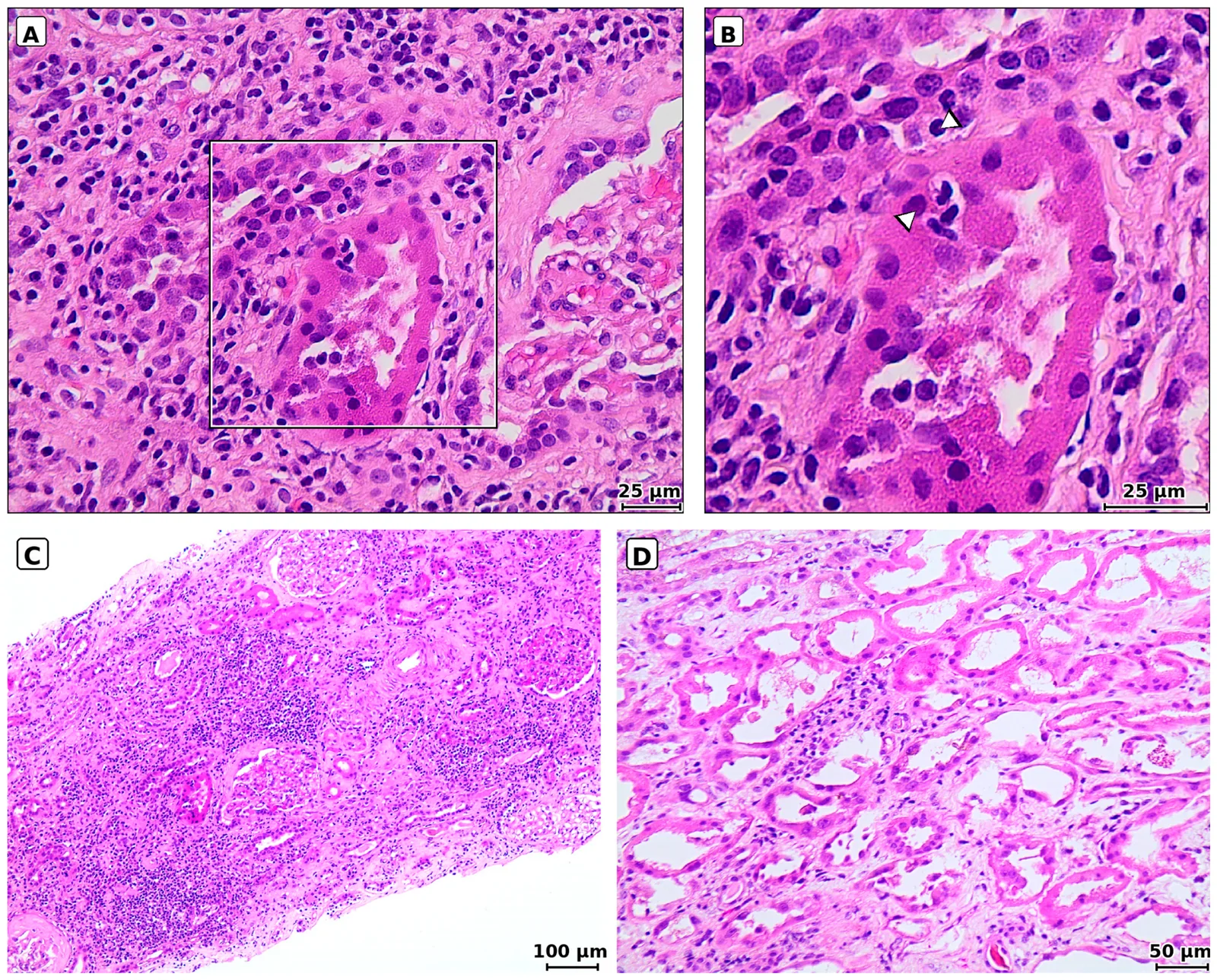
Representative renal histopathologic findings in biopsy-confirmed acute interstitial nephritis clinically attributed to immune checkpoint inhibitor exposure. (**A**) Hematoxylin and eosin (H&E)-stained renal biopsy showing a dense interstitial inflammatory infiltrate surrounding a renal tubule; the boxed region is enlarged in panel (**B**) (scale bar, 25 μm). (**B**) Digital enlargement of the boxed region in panel (**A**), showing inflammatory cells infiltrating the tubular epithelium, consistent with tubulitis (arrowheads); the scale bar was derived from the calibrated scale in panel (**A**) (scale bar, 25 μm). (**C**) Dense lymphocytic interstitial infiltrates in a background of moderate-to-severe interstitial fibrosis and tubular atrophy (H&E; scale bar, 100 μm). (**D**) Acute tubular injury characterized by tubular dilatation, epithelial flattening, epithelial sloughing, and regenerative epithelial changes, accompanied by interstitial edema (H&E; scale bar, 50 μm). (Panels **A**,**B**) are from the same biopsy; (panels **C**,**D**) are from two additional representative biopsies from the cohort. Images were cropped and resized for panel assembly; the box, arrowheads, scale bars, and scale-bar labels were added or enhanced as annotations. No other digital image manipulation was performed.

IFTA was classified as mild in three patients, moderate in eight patients, and severe in one patient. The extent of global glomerulosclerosis varied, with five patients having global glomerulosclerosis involving ≥20% of sampled glomeruli. Immunofluorescence showed no diagnostically significant immune deposits in three patients, trace or nonspecific IgM-predominant staining in six, trace or nonspecific C3-predominant staining in two, and combined IgA/C3 staining in one. In one biopsy categorized as showing no diagnostic immune-complex pattern, only trace granular mesangial IgM and C3 were reported, with IgG, IgA, C1q, and κ and λ light chains negative. Electron microscopy was not consistently available and was therefore not summarized as a cohort-level negative finding. Data were available for all variables displayed in Table 4. Representative interstitial inflammation, tubulitis, chronic tubulointerstitial change, and acute tubular injury are shown in Figure 1, and selected descriptive pathology characteristics are summarized in Table 4. Individual renal histopathologic findings are provided in Table S2.

The two patients with marked microscopic hematuria (20–25 and 100 red blood cells per high-power field) were evaluated in their clinicopathologic context because this degree of hematuria may indicate a competing glomerular or bleeding-related process. One patient had IgA 2+ and C3 2+ staining on immunofluorescence. However, proteinuria was subnephrotic, and the available pathology report did not identify an active proliferative, necrotizing, or crescentic glomerular lesion as the dominant abnormality. The other patient had marked microscopic hematuria, negative immunofluorescence findings, and epistaxis at presentation. In both patients, the final clinicopathologic diagnosis remained AIN, and no alternative glomerular disorder was considered the principal cause of AKI.

### 3.6. Exploratory Pathology–Outcome Analyses

Exploratory descriptive assessment did not identify a consistent pattern between chronic histopathologic changes and renal recovery. Moderate or severe IFTA was present in 7 of 8 patients with complete recovery and 2 of 4 patients with incomplete recovery; global glomerulosclerosis involving ≥20% of sampled glomeruli was present in 4 of 8 and 1 of 4 patients, respectively. These unadjusted counts are descriptive only. Given the small cohort, very small and imbalanced subgroups, differences in baseline renal function, and retrospective pathology grading, no prognostic inference was made.

## 4. Discussion

In this single-center retrospective case series, we characterized 12 patients with biopsy-confirmed AIN clinically attributed to immune checkpoint inhibitor exposure (ICI-AIN). ICI-AIN accounted for 3.5% of native renal biopsies and was detected in 0.8% of unique ICI-treated patients at our institution. The cohort consisted predominantly of patients with lung cancer, and most reached KDIGO stage 3 during the AKI episode. AKI was detected during routine laboratory monitoring in all cases; clinical and urinary findings were nonspecific, and renal recovery at the last available assessment was complete in eight patients, partial in two, and absent in two according to the predefined criteria.

AKI in patients receiving ICIs has numerous competing causes, including acute tubular injury, hemodynamic or obstructive injury, infection, contrast, chemotherapy, and other medications [8,9]. Biopsy established AIN but not the responsible exposure. Seven patients were receiving acid-suppressive therapy, and the available records did not permit uniform exclusion of other potential contributors [5,8]. Thus, ICI-AIN denotes a clinicopathologic attribution rather than histology-specific proof of ICI causation.

The high proportion of patients reaching KDIGO stage 3 likely reflects real-world biopsy selection, because severe, persistent, or diagnostically uncertain AKI is more likely to undergo histopathologic evaluation [12,14].

The median 27.5-day interval from AKI onset to biopsy likely reflects evaluation of alternative etiologies, nephrology consultation, and procedural logistics. The 152-day outlier followed initial procedural deferral and persistent diagnostic uncertainty. Because the timing of corticosteroid initiation was unavailable and the outlier was singular, biopsy timing should not be interpreted as treatment delay or used to infer effects on histologic chronicity or renal outcome.

The clinical presentation of ICI-AIN was largely nonspecific. All cases were detected during routine laboratory monitoring, and only three patients had additional symptoms or signs at presentation. Urinary findings were compatible with AIN but were not diagnostic. Pyuria was present in all patients, and urine cultures were negative in all cases; hematuria occurred in a minority, proteinuria was uniformly subnephrotic, and peripheral eosinophilia was uncommon. These findings underscore the value of urinalysis as part of the evaluation of AKI in ICI-treated patients, while emphasizing that urinary abnormalities cannot reliably confirm or exclude ICI-AIN.

Marked microscopic hematuria highlights the importance of histopathologic evaluation when urinary findings are atypical. IgA/C3 staining may reflect incidental immune-complex deposition or a concurrent glomerular process; however, no active proliferative, necrotizing, or crescentic glomerular lesion was identified as the predominant abnormality, and AIN remained the principal clinicopathologic diagnosis. Concurrent glomerular disease cannot be completely excluded in a retrospective biopsy series and should be considered when urinary findings are atypical for isolated tubulointerstitial disease.

Acute tubular injury coexisted with AIN in 7 of 12 biopsies. Because tubular epithelial injury can accompany interstitial inflammation but may also reflect hemodynamic, ischemic, chemotherapeutic, or other nephrotoxic insults, this finding supports a potentially multifactorial AKI phenotype rather than drug-specific histologic causality. In each case, however, AIN remained the final dominant clinicopathologic diagnosis.

The role of renal biopsy in suspected ICI-associated renal injury remains a matter of clinical debate. Oncology-oriented guidance generally emphasizes the exclusion of alternative causes and may support empiric treatment in selected patients, whereas nephrology-oriented approaches place greater emphasis on histopathologic confirmation when the diagnosis is uncertain, urinary findings are atypical, AKI is severe or persistent, or treatment decisions have important renal and oncologic implications [12,13,14,18]. Our findings are consistent with an individualized and selective approach. Renal biopsy is not required in every ICI-treated patient who develops AKI, but it can provide valuable diagnostic information when alternative etiologies are plausible or when the decision to interrupt immunotherapy or initiate high-dose corticosteroids is clinically consequential.

The two institutional proportions use different denominators: 12/339 (3.5%) among native renal biopsies and 12/1472 (0.8%) among unique ICI-treated patients. Because ascertainment required clinical recognition and biopsy, these values describe detection within institutional practice, not population incidence. Lung cancer predominance may reflect local ICI use rather than tumor-specific susceptibility.

No patient required kidney replacement therapy, but residual dysfunction was common (last eGFR, 30–79 mL/min/1.73 m^2^). Under the primary definition, recovery was complete in eight patients (66.7%), partial in two (16.7%), and absent in two (16.7%); the latter had 42.5% and 38.8% decreases from peak SCr. Under the sensitivity definition, five patients (41.7%) had complete, five (41.7%) partial, and two (16.7%) absent recovery. Three patients with low baseline SCr were reclassified from complete to partial recovery, showing that an absolute SCr threshold may overstate return to baseline. The criteria were adapted from a biopsy-confirmed AIN study [17], but comparison is limited because that study used a fixed 6-month assessment, whereas ours used the last available measurement.

Exploratory review did not identify a consistent relationship between chronic histopathologic changes and renal recovery. However, the cohort and individual pathology subgroups were too small and imbalanced to support prognostic interpretation, and these observations should not be taken as evidence for or against an association between chronicity and outcome.

Corticosteroids remain the principal treatment for AIN clinically attributed to ICI exposure, but the optimal regimen remains uncertain [13,14]. All patients received corticosteroids, most often intravenous methylprednisolone pulses followed by an oral taper. The small cohort and incomplete treatment data precluded evaluation of timing or dose–response relationships. Observational studies have associated earlier initiation with improved renal recovery, although confounding remains [9], and recent evidence suggests that shorter courses may be feasible in selected patients [19].

Limitations include the retrospective, single-center, biopsy-selected design and small cohort. Biopsy decisions were not protocolized, limiting generalizability to the full spectrum of renal lesions in ICI-treated patients. Clinical attribution was not independently adjudicated, and competing causes could not be excluded uniformly. Incomplete exposure chronology and corticosteroid-treatment data precluded analyses of exposure timing or treatment–response relationships. Histopathologic variables came from routine reports rather than blinded re-review, separately reported glomerular counts were inconsistent, and electron microscopy was not uniformly available. AKI staging used peak SCr because urine-output data were unavailable. Follow-up intervals and SCr measurement frequency varied, and death limited observation; recovery times therefore represent first documented threshold attainment, and recovery categories were assessed at nonuniform last follow-up.

Strengths include biopsy confirmation and integration of patient-level clinical, laboratory, urinary, histopathologic, and available treatment data. Defined native renal biopsy and ICI-treated-patient denominators also provided complementary institutional estimates across renal biopsy practice and the broader treated population.

In conclusion, biopsy-confirmed AIN clinically attributed to ICI exposure was detected infrequently among native renal biopsies and ICI-treated patients at our institution. Most patients reached KDIGO stage 3 during the episode, although AKI was initially detected during routine laboratory monitoring. No patient required kidney replacement therapy, but residual renal dysfunction was common; the lowest eGFR at the last available assessment was 30 mL/min/1.73 m^2^, and two patients did not meet the prespecified partial-recovery criterion. These findings underscore the importance of monitoring kidney function, the value of urinalysis in evaluating ICI-associated AKI, and the need for careful assessment of competing causes. Close collaboration between nephrologists and oncologists and selective renal biopsy may be appropriate when AKI is unexplained, persistent, diagnostically uncertain, or likely to influence important renal or oncologic treatment decisions.

## Figures and Tables

**Table 1:** Baseline demographic, renal, oncologic, treatment, and biopsy-timing characteristics of patients with biopsy-confirmed AIN clinically attributed to ICI exposure.

Characteristic	Overall Cohort (*n* = 12)
Demographic characteristics	
Age at biopsy, years	70 (63–73)
Male sex	10 (83.3)
Baseline renal function	
Serum creatinine, mg/dL	1.05 (0.88–1.13)
eGFR, mL/min/1.73 m^2^	74 (62–96)
Baseline eGFR < 60 mL/min/1.73 m^2^ *	3 (25.0)
Underlying malignancy	
Lung cancer	10 (83.3)
Lung adenocarcinoma	7 (58.3)
NSCLC, not otherwise specified	2 (16.7)
SCLC	1 (8.3)
Malignant pleural mesothelioma	1 (8.3)
Esophageal adenocarcinoma	1 (8.3)
ICI regimen	
Pembrolizumab	5 (41.7)
Nivolumab plus ipilimumab	3 (25.0)
Atezolizumab	2 (16.7)
Nivolumab	1 (8.3)
Durvalumab	1 (8.3)
Concomitant medications	
Proton pump inhibitor	3 (25.0)
Histamine H_2_-receptor antagonist	4 (33.3)
Clinical presentation and biopsy timing	
AKI detected by routine laboratory monitoring	12 (100.0)
Additional clinical manifestations	3 (25.0)
Time from AKI onset to biopsy, days	27.5 (16–30)

Note: Data are presented as median (interquartile range) or *n* (%). All percentages, including those for lung cancer histologic subtypes, are based on the full cohort (*n* = 12). * Baseline eGFR < 60 mL/min/1.73 m^2^ is a descriptive single-value category and is not equivalent to a confirmed diagnosis of chronic kidney disease without evidence of chronicity. Additional clinical manifestations were lower-extremity edema, fatigue or malaise, and epistaxis (one patient each). Abbreviations: AKI, acute kidney injury; eGFR, estimated glomerular filtration rate; ICI, immune checkpoint inhibitor; ICI-AIN, acute interstitial nephritis clinically attributed to immune checkpoint inhibitor exposure; NSCLC, non-small cell lung cancer; SCLC, small-cell lung cancer.

**Table 2:** Baseline renal function, laboratory findings at biopsy assessment, peak serum creatinine, and urinary findings in patients with biopsy-confirmed AIN clinically attributed to ICI exposure.

Patient	Baseline SCr (mg/dL)	SCr at Biopsy Assessment (mg/dL)	Peak SCr (mg/dL)	KDIGO AKI Stage	WBCs/HPF	RBCs/HPF	24-h Urinary Protein Excretion (mg/24 h)	Peripheral Eosinophilia
1	1.70	2.50	2.50	1	5–10	0–2	670	No
2	1.69	3.80	4.10	3	7–8	3–4	200	No
3	0.74	2.17	2.90	3	6–7	0–2	163	Yes
4	0.90	3.80	6.80	3	10–15	0–2	350	No
5	0.90	2.80	4.60	3	10–15	3–4	144	No
6	0.80	3.70	3.80	3	10–15	0–2	300	No
7	1.10	4.80	4.80	3	5–6	0–2	190	No
8	1.10	3.30	3.30	3	5–10	0–2	220	No
9	1.00	4.50	5.42	3	5–6	20–25	240	No
10	1.20	4.10	4.30	3	20	100	81	Yes
11	1.10	1.88	2.59	2	6–7	0–2	47	No
12	0.70	1.80	2.45	3	5–6	0–2	335	No

**Notes**: SCr at biopsy assessment was the value obtained on the biopsy date or the closest preceding value during the same AKI episode. Pyuria was defined as ≥5 WBCs/HPF, hematuria as >2 RBCs/HPF, and peripheral eosinophilia as an absolute eosinophil count >500 cells/µL. Urine cultures were negative in all 12 patients. A 24-h urinary protein measurement was available for all 12 patients; all values were subnephrotic. AKI stage was assigned using peak SCr during the AKI episode according to KDIGO serum creatinine criteria. The reported stage therefore reflects the highest stage reached during the episode and should not be interpreted as the stage at renal biopsy. Median peak SCr was 3.95 mg/dL (IQR, 2.82–4.65); KDIGO stage 1, 2, and 3 AKI occurred in 1 (8.3%), 1 (8.3%), and 10 (83.3%) patients, respectively. **Abbreviations**: AKI, acute kidney injury; HPF, high-power field; ICI-AIN, acute interstitial nephritis clinically attributed to immune checkpoint inhibitor exposure; IQR, interquartile range; KDIGO, Kidney Disease: Improving Global Outcomes; RBCs, red blood cells; SCr, serum creatinine; WBCs, white blood cells.

**Table 3:** Renal-function outcomes at the last available assessment and vital status at last follow-up in patients with biopsy-confirmed AIN clinically attributed to ICI exposure.

Patient	SCr at Last Assessment (mg/dL)	eGFR at Last Assessment (mL/min/1.73 m^2^)	Primary Renal Recovery Classification	Sensitivity Classification (Stricter Complete- Recovery Criterion)	Time to Assigned Recovery Endpoint (Days)	Vital Status
1	1.86	41	Complete	Complete	210	Alive
2	2.00	30	Complete	Complete	358	Deceased
3	1.00	79	Complete	Partial	58	Alive
4	1.35	59	Complete	Partial	62	Alive
5	1.33	60	Complete	Partial	57	Alive
6	1.90	38	Partial	Partial	63	Deceased
7	1.30	58	Complete	Complete	117	Deceased
8	1.18	49	Complete	Complete	31	Alive
9	1.90	36	Partial	Partial	58	Deceased
10	1.24	61	Complete	Complete	117	Alive
11	1.49	45	Absent	Absent	Not reached	Alive
12	1.50	51	Absent	Absent	Not reached	Alive

Notes: Under the primary definition, complete recovery was defined as SCr returning to within 25% of baseline or to <1.4 mg/dL. Partial recovery was defined as a ≥50% decrease from peak SCr without meeting complete-recovery criteria, calculated as [(peak SCr − assessment SCr)/peak SCr] × 100; absent recovery indicated failure to meet complete- or partial-recovery criteria or persistent kidney replacement therapy dependence. Under the sensitivity definition, complete recovery required SCr to return to within 25% of baseline; the same partial- and absent-recovery criteria were applied. Time to the assigned recovery endpoint was calculated according to the primary recovery classification. Time to the assigned recovery endpoint denotes the interval from AKI onset to the first documented SCr measurement meeting complete- or partial-recovery criteria; “Not reached” indicates that neither endpoint was met during available follow-up. Patients 11 and 12 had 42.5% and 38.8% reductions from peak SCr, respectively, and were therefore classified as absent recovery. All patients received corticosteroids; patient 7 also received mycophenolate mofetil. No patient required kidney replacement therapy. All four deaths were attributed to the underlying malignancy. Abbreviations: eGFR, estimated glomerular filtration rate; ICI-AIN, acute interstitial nephritis clinically attributed to immune checkpoint inhibitor exposure; SCr, serum creatinine.

**Table 4:** Selected renal histopathologic findings in patients with biopsy-confirmed AIN clinically attributed to ICI exposure.

Characteristic	Overall Cohort (*n* = 12)
Acute findings	
Acute interstitial nephritis	12 (100.0)
Acute tubular injury	7 (58.3)
Interstitial fibrosis and tubular atrophy	
Mild	3 (25.0)
Moderate	8 (66.7)
Severe	1 (8.3)
Global glomerulosclerosis	
<20% of sampled glomeruli	7 (58.3)
≥20% of sampled glomeruli	5 (41.7)
Immunofluorescence findings	
No diagnostically significant immune deposits	3 (25.0)
Trace/nonspecific IgM-predominant staining	6 (50.0)
Trace/nonspecific C3-predominant staining	2 (16.7)
Combined IgA/C3 staining	1 (8.3)

Note: Percentages are based on the full cohort (*n* = 12). Immunofluorescence categories reflect the principal reported pattern in each biopsy and distinguish trace or nonspecific staining from a diagnostic immune-complex glomerular pattern. Unavailable or unreported electron microscopy was not interpreted as negative. Pathologic features not described in routine reports were treated as unavailable rather than absent unless their absence was explicitly stated. Abbreviations: C3, complement component 3; ICI-AIN, acute interstitial nephritis clinically attributed to immune checkpoint inhibitor exposure; IgA, immunoglobulin A; IgM, immunoglobulin M.

## Data Availability

Selected de-identified patient-level demographic, clinical, biopsy-timing, and histopathological variables are provided in Supplementary Tables S1 and S2. The underlying source medical records, exact dates, and potentially identifying clinical information are not publicly available because they are subject to privacy and ethical restrictions. Additional de-identified data may be available from the corresponding author upon reasonable request and subject to institutional approval.

## Supplementary Materials

The following supporting information can be downloaded at: https://www.mdpi.com/article/10.3390/antib15040065/s1, Table S1: Individual baseline demographic, renal, oncologic, clinical, and biopsy-timing characteristics of patients with biopsy-confirmed AIN clinically attributed to ICI exposure; Table S2: Individual renal histopathologic findings in patients with biopsy-confirmed AIN clinically attributed to ICI exposure.

## Abbreviations

The following abbreviations are used in this manuscript:

AIN	acute interstitial nephritis
AKI	acute kidney injury
C1q	complement component 1q
C3	complement component 3
CKD-EPI	Chronic Kidney Disease Epidemiology Collaboration
CTLA-4	cytotoxic T-lymphocyte-associated antigen 4
eGFR	estimated glomerular filtration rate
HPF	high-power field
ICI	immune checkpoint inhibitor
ICI-AIN	acute interstitial nephritis clinically attributed to immune checkpoint inhibitor exposure
ICI-AKI	immune checkpoint inhibitor-associated acute kidney injury
IFTA	interstitial fibrosis and tubular atrophy
IgA	immunoglobulin A
IgG	immunoglobulin G
IgM	immunoglobulin M
IQR	interquartile range
KDIGO	Kidney Disease: Improving Global Outcomes
NSCLC	non-small cell lung cancer
PD-1	programmed cell death protein 1
PD-L1	programmed death-ligand 1
RBCs	red blood cells
SCLC	small-cell lung cancer
SCr	serum creatinine
WBCs	white blood cells
