# Supplementary material for: Biopsy-Confirmed Acute Interstitial Nephritis in Patients Treated with Immune Checkpoint Inhibitors: A Single-Center Retrospective Case Series
Source: Antibodies (Basel). 2026 Jul 27;15(4):65. doi: 10.3390/antib15040065 (PMC13509303; doi:10.3390/antib15040065)
Supplement: Supplementary file 1 [file antibodies-15-00065-s001.zip › antibodies-4396390-supplementary.pdf]

Supplementary Tables

Biopsy-Confirmed Acute Interstitial Nephritis in Patients Treated with Immune Checkpoint Inhibitors: A Single-Center Retrospective Case Series

Table S1. Individual baseline demographic, renal, oncologic, clinical, and biopsy-timing characteristics of patients with biopsy-confirmed AIN clinically attributed to ICI exposure.

Patient	Sex	Age, years	Baseline SCr, mg/dL	Baseline eGFR, mL/min/1.73 m^2	ICI regimen	Malignancy and histology	Additional clinical manifestation	Time from AKI onset to biopsy, days
1	Male	58	1.70	46	Pembrolizumab	Lung adenocarcinoma	Lower-extremity edema	152
2	Female	51	1.69	36	Nivolumab plus ipilimumab	Malignant pleural mesothelioma	Fatigue or malaise	27
3	Male	73	0.74	96	Nivolumab plus ipilimumab	NSCLC, not otherwise specified	None	5
4	Male	63	0.90	96	Nivolumab plus ipilimumab	Lung adenocarcinoma	None	26
5	Male	62	0.90	97	Nivolumab monotherapy	SCLC	None	31
6	Male	69	0.80	96	Pembrolizumab	Lung adenocarcinoma	None	28
7	Male	74	1.10	70	Pembrolizumab	Esophageal adenocarcinoma	None	92
8	Female	71	1.10	54	Atezolizumab	Lung adenocarcinoma	None	29
9	Male	77	1.00	78	Pembrolizumab	Lung adenocarcinoma	None	18
10	Male	73	1.20	64	Durvalumab	NSCLC, not otherwise specified	Epistaxis	10
11	Male	86	1.10	65	Pembrolizumab	Lung adenocarcinoma	None	7
12	Male	67	0.70	101	Atezolizumab	Lung adenocarcinoma	None	28

All patients had AKI identified through laboratory monitoring. Abbreviations: AKI, acute kidney injury; eGFR, estimated glomerular filtration rate; ICI, immune checkpoint inhibitor; ICI-AIN, acute interstitial nephritis clinically attributed to immune checkpoint inhibitor exposure; NSCLC, non–small cell lung cancer; SCLC, small-cell lung cancer; SCr, serum creatinine. Patient numbers are study identifiers and do not correspond to medical record numbers; direct identifiers, exact dates, and protocol numbers are not included.

Table S2. Individual renal histopathologic findings in patients with biopsy-confirmed AIN clinically attributed to ICI exposure.

Patient	Acute tubular injury	Global glomerulosclerosis, % of sampled glomeruli	IFTA	Immunofluorescence finding
1	Yes	10	Moderate	C3+
2	No	30	Moderate	IgM+
3	Yes	10	Moderate	Trace mesangial IgM and C3; no diagnostically significant immune-complex pattern.
4	Yes	20	Mild	IgM+
5	No	16	Moderate	IgM++
6	Yes	10	Mild	Negative
7	No	12	Moderate	C3+
8	Yes	30	Moderate	IgM+
9	No	14	Severe	IgA++/C3++
10	Yes	20	Moderate	Negative
11	Yes	50	Moderate	IgM+
12	No	10	Mild	IgM+

All biopsies showed interstitial inflammatory infiltrates consistent with acute interstitial nephritis. The symbols + and ++ reproduce the semiquantitative intensity notation used in the original pathology reports. Abbreviations: C3, complement component 3; ICI-AIN, acute interstitial nephritis clinically attributed to immune checkpoint inhibitor exposure; IFTA, interstitial fibrosis and tubular atrophy; IgA, immunoglobulin A; IgM, immunoglobulin M.
